# Topoisomerase IB poisons induce histone H2A phosphorylation as a response to DNA damage in *Leishmania infantum*

**DOI:** 10.1016/j.ijpddr.2019.09.005

**Published:** 2019-09-20

**Authors:** Camino Gutiérrez-Corbo, Raquel Álvarez-Velilla, Rosa M. Reguera, Carlos García-Estrada, Mark Cushman, Rafael Balaña-Fouce, Yolanda Pérez-Pertejo

**Affiliations:** a Departamento de Ciencias Biomédicas, Universidad de León, Campus de Vegazana S/n, 24071, León, Spain; b INBIOTEC (Instituto de Biotecnología de León), Avda. Real 1 - Parque Científico de León, 24006, León, Spain; c Department of Medicinal Chemistry, and Molecular Pharmacology, College of Pharmacy, The Purdue Center for Cancer Research, Purdue University, Lafayette, IN, USA

**Keywords:** Leishmania, DNA-Topoisomerase IB, Camptothecin, Indenoisoquinolines, Histone H2A, DNA damage

## Abstract

DNA topoisomerases are considered consolidated druggable targets against diseases produced by trypanosomatids. Several reports indicated that indenoisoquinolines, a family of non-camptothecinic based topoisomerase poisons, have a strong leishmanicidal effect both *in vitro* and *in vivo* in murine models of visceral leishmaniasis. The antileishmanial effect of the indenoisoquinolines implies several mechanisms that include the stabilization of the cleavage complex, histone H2A phosphorylation and DNA fragmentation.

A series of 20 compounds with the indenoisoquinoline scaffold and several substituents at positions N6, C3, C8 and C9, were tested both in promastigotes and in intramacrophage splenic amastigotes obtained from an experimental murine infection. The antileishmanial effect of most of these compounds was within the micromolar or submicromolar range. In addition, the introduction of an N atom in the indenoisoquinoline ring (7-azaindenoisoquinolines) produced the highest selectivity index along with strong DNA topoisomerase IB inhibition, histone H2A phosphorylation and DNA-topoisomerase IB complex stabilization.

This report shows for the first time the effect of a series of synthetic indenoisoquinolines on histone H2A phosphorylation, which represents a primary signal of double stranded DNA break in genus *Leishmania*.

## Highlights

• N-6 indenoisoquinoline derivatives show strong antileishmanial activity.
• Indenoisoquinolines arrest *Leishmania* cell-cycle in S phase.
• Inhibition of leishmanial TopIB by indenoisoquinolines induces DNA fragmentation.
• Leishmanial H2A histone is phosphorylated at the Thr128 in response to DNA damage.

## Graphical abstract

**Figure:**
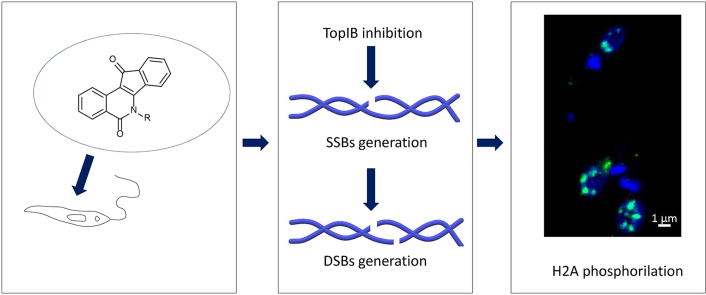

## Abbreviations

Visceral Leishmaniasis: VL
pentavalent antimony: SbV
DNA topoisomerases: Top
DNA topisomerase IB: TopIB
Leishmania topoisomerase IB: LTopIB
human Top IB: hTopIB
camptothecin: CPT
topotecan: TPT
single-strand breaks: SSB
double-strand break: DSB
phosphatidylinositol-3-OH-kinase-like family: PIKKs
Ataxia Telangiectasia Mutated: ATM
Ataxia Telangiectasia-Rad3 related: ATR
heat-inactivated fetal bovine serum: FBS
phosphate-buffered saline: PBS
Selectivity Index: SI
4,6-diamino-2-phenylindole: DAPI
Tyrosyl DNA-phosphodiesterase: Tdp1

## 1 Introduction

Visceral leishmaniasis (VL) is a serious zoonotic disease caused by parasites of Gen. *Leishmania*, which is responsible for 20,000 to 30,000 deaths every year mostly in Africa (Alvar et al., 2012; Burza et al., 2018). First-choice drugs are based on organic complexes of pentavalent antimony (Sb^V^), which are losing effectiveness due to excessive use and lack of replacement (Frézard et al., 2009; Bush et al., 2017). A single dose of a liposomal suspension of amphotericin B (AmBisome) (Sundar et al., 2010), and the oral drug miltefosine (Bhattacharya et al., 2007), are being used as second-line treatments, either alone or in combination with Sb^V^ (Kimutai et al., 2017). However, in the case of AmBisome, its use is limited due to, poor chemical stability at the point of care and its mandatory intravenous route of administration (Stone et al., 2016; Jensen, 2017). In the case of miltefosine, teratogenic concerns prevent its administration to pregnant women, and pregnancy should be avoided the next 4 months after the end of the miltefosine treatment (Dorlo et al., 2012). Therefore, the urgency of new antileishmanial drugs based on validated objectives is a real need, especially when big pharmaceutical companies are recently committed to invest more funds and efforts in the development of novel treatments.

Control of DNA topology by DNA topoisomerases (Top) is considered a consolidated target for drug development in cancer and infectious diseases, including leishmaniasis (Champoux, 2001; Pommier et al., 2016). The heterodimeric *Leishmania* topoisomerase IB (LTopIB) is structurally dissimilar from the monomeric human Top IB (hTopIB), which makes it interesting as a therapeutic target (Balaña-Fouce et al., 2014; Chowdhury and Majumder, 2019; Velásquez et al., 2017). Mechanistically, DNA topoisomerase IB (TopIB) cleaves one of the DNA strands, establishing a reversible phosphodiester bond to the 3′-end and releasing a 5′-OH at the free end (Stewart et al., 1998). This step is particularly dramatic, since the intermediate can be stabilized by certain compounds called topoisomerase poisons, which trap the covalent TopIB-DNA cleavage complex, thus producing single-strand DNA breaks (SSB) (Pommier et al., 2010). TopIB poisons, such as camptothecin (CPT) (a natural product from the Chinese tree *Camptotheca accuminata*) and non-CPT compounds such as the synthetic indenoisoquinolines, are in the pipeline of anticancer drugs, and several reports have shown their efficacy in preclinical models of VL (Balaña-Fouce et al., 2012; Pommier, 2013; Pommier et al., 2015). Two indenoisoquinolines, LMP400 (indotecan) and LMP776 (indimitecan), have completed phase 1 clinical trials (NCT01051635; https://clinicaltrials.gov/ct2/show/NCT01051635), and a third indenoisoquinoline, LMP744, has recently started phase 1 clinical trials (NCT03030417; https://clinicaltrials.gov/ct2/show/NCT03030417). Indenoisoquinolines have several advantages over CPT derivatives, including i) higher chemical stability; ii) increased persistence of the ternary drug-DNA-TopIB cleavage complex; and iii) ability to overcome multidrug resistance systems (Antony et al., 2007). It is well-known that SSBs produced by these compounds can progress to double-strand breaks (DSBs) when the replication fork collides with them during DNA replication (Hsiang et al., 1985, 1989). DSBs may compromise genomic stability and cell viability in eukaryotic cells. In mammalian cells, an early molecular response to DSBs generation is the rapid phosphorylation of the histone H2AX on Ser^139^, which is called γH2AX. This process is mediated by members of phosphatidylinositol-3-OH-kinase-like family (PIKKs), Ataxia Telangiectasia Mutated (ATM), Ataxia Telangiectasia-Rad3 related (ATR) and DNA-dependent protein kinases (DNA-PKcs) (Kinner et al., 2008). However, genetic studies carried out in *T. brucei* showed that Ser^139^ is substituted by a Thr residue in the histone H2A, which is phylogenetically conserved in *Leishmania* spp. and it is susceptible of phosphorylation (Glover and Horn, 2012). The generation of γH2AX foci has been observed within minutes after exposure to TopIB poisons, and has been used to monitor the activity of CPT derivatives and indenoisoquinolines in human cells (Huang et al., 2004; Patel et al., 2016).

Previous studies have shown that CPT derivatives and indenoisoquinolines are potent antileishmanial compounds with good selectivity indexes *vs.* murine macrophages (Balaña-Fouce et al., 2012). It is very likely that exposure to these compounds can initiate phosphorylation of Thr in histone H2A to generate γH2A prior to the onset of DNA repair mechanisms. Previous studies showed that *T. brucei* and other kinetoplastids were able to phosphorylate H2A histone after a DNA insult produced by exposure to chemical or enzymatic DNA damage. γH2A foci colocalize with RAD51 repair foci and they were observed mostly in nuclei in S-phase and G2 (Glover and Horn, 2012). Besides, the phosphorylation of H2A histone in this amino acid has been used as signal of DNA damage in *L. maj*or, studying different enzymes involved in the DNA damage response in this parasite (Damasceno et al., 2016, 2018).

The current work describes the antileishmanial activity of a group of indenoisoquinolines against both stages of *L. infantum*. The cytotoxicity profiles of these compounds have been compared to their activity as TopIB poisons. In addition, leishmanial H2A histone phosphorylation on a putative phosphorylation site (Thr^128^) has been studied for the first time as a DNA damage signal produced by TopIB inhibitors.

## 2 Material and methods

### 2.1 Reagents

Cell culture media, CPT, SN38, wortmannin and caffeine were purchased from Sigma-Aldrich (Spain). Topotecan (TPT) (Hycamtin®) was obtained from GlaxoSmithKline. Indenoisoquinolines (compounds **1** to **20)** were kindly provided by Dr. Mark Cushman (Department of Medicinal Chemistry, Purdue University, Indiana, USA). The KLH-conjugated phospho-peptide KKGKA [pT]PSA, epitope of *L. infantum* histone H2A (Glover and Horn, 2012), was synthetized by Open BioSystems (GE Healthcare). Polyclonal anti-γH2A antiserum was obtained for the KLH-conjugated KKGKA [pT]PSA phosphopeptide in New Zealand White male rabbits housed in the Animal House facility of University of León (Spain), and according to a standard 90-day protocol.

### 2.2 *Leishmania* strains

Histone phosphorylation assays were performed in promastigotes of *L. infantum* BCN 150 strain, whereas the antiparasitic activity of the compounds was assessed on the transgenic strain that constitutively expresses the irfp gene encoding the infrared iRFP protein (iRFP *L. infantum* strain) (Calvo-Álvarez et al., 2015), which is derived from *L. infantum* BCN 150. Both strains were grown at 26 °C in M-199 medium (Gibco) supplemented with 25 mM HEPES pH 7.2, 0.1 mM adenine, 0.0005% (w/v) hemin, 2 μg/mL biopterin, 0.0001% (w/v) biotin, 10% (v/v) heat-inactivated fetal bovine serum (FBS) and an antibiotic cocktail comprising 50 U/mL penicillin and 50 μg/mL streptomycin.

### 2.3 *Ex vivo* murine splenic explant cultures

All protocols described in this work were approved by the Animal Care Committee of the University of Leon, project license SAF2017-83575-R. It complies with European Union Legislation (2010/63/UE) and Spanish Act (RD 53/2013).

Five-week BALB/c female mice infected intraperitoneally with 1.5 × 10^9^ metacyclic iRFP *L. infantum* promastigotes were sacrificed to eviscerate their spleens, which were processed to obtain a suspension of primary splenocytes [45]. Briefly, freshly dissected spleens were washed with cold phosphate-buffered saline (PBS), cut in small pieces and incubated for 20 min with 5 mL of 2 mg/mL collagenase D (Sigma) prepared in buffer (10 mM HEPES, pH 7.4, 150 mM NaCl, 5 mM KCl, 1 mM MgCl_2_ and 1.8 mM CaCl_2_). Then, the cell suspension was passed through a 100 μm cell strainer, harvested by centrifugation (500×*g* for 7 min at 4 °C), washed twice with PBS, and resuspended in RPMI medium (Gibco) supplemented with 10 mM HEPES, 1 mM sodium pyruvate, 1xRPMI 1640 vitamin mix, 10% (v/v) FBS, 50 U/mL penicillin and 50 μg/mL streptomycin. The cell suspension was cultured in 384-well optical bottom black plates (Thermo Scientific) at 37 °C under 5% CO_2_ atmosphere.

### 2.4 Determination of the antileishmanial and cytotoxic effects

The antileishmanial activity of the compounds was tested both in promastigotes and in amastigotes infecting mouse splenocytes. For this purpose, 384-well optical bottom black plates (Thermo Scientific) were seeded either with free-living iRFP *L. infantum* promastigotes or with splenic explants infected with iRFP *L. infantum* amastigotes, which were incubated at 26 °C or 37 °C, respectively. The viability of both iRFP *L. infantum* stages was measured by recording the fluorescence emitted by viable cells at 708 nm in an Odyssey (Li-Cor) infrared imaging system after a 72-h period of incubation with different concentrations of the compounds. Dose-response curves were obtained by plotting the infrared fluorescence emission of viable parasites *vs.* different concentrations of testing compounds and were fitted by nonlinear analysis using the Sigma-Plot 10.0 statistical package. The reduction of infrared emission with respect to the negative control was used to determine the 50% effective concentration to kill the parasites (EC_50_). Both controls and treated groups were tested with DMSO (drug vehicle) concentrations below 0.1%. All compounds and controls were assayed in triplicate.

Finally, the cytotoxicity of the compounds was assessed on freshly-isolated mouse splenocytes, which were obtained from uninfected BALB/c mice according to the protocol previously described. Viability of uninfected splenocytes was used to determine the cytotoxic concentration 50 (CC_50_) by means of the Alamar Blue staining method, according to manufacturer's recommendations (Invitrogen). Selectivity index (SI) for each compound was calculated as the ratio between the CC_50_ value obtained for splenic cells and the EC_50_ value for amastigotes.

### 2.5 DNA topoisomerase IB assays

Expression and purification of LTopIB and hTopIB were carried out according to a previously standardized protocol (Villa et al., 2003). The effect of indenoisoquinolines (compounds **1** to **20**) on recombinant LTopIB and hTopIB was determined by measuring the relaxation of negatively supercoiled pBluescript-SK DNA plasmid (pSK). Briefly, in a total volume of 20 μL, 100 U of recombinant TopIB were incubated with 0.5 μg of pSK DNA in 10 mM Tris-HCl buffer (pH 7.5), 5 mM MgCl_2_, 5 mM dithiothreitol (DTT), 0.1 mM EDTA, 15 mg/mL bovine serum albumin, and 150 mM KCl. Different concentrations of indenoisoquinolines were also added. Following a 4-min incubation period at 25 °C, reactions were stopped with 1% (w/v) SDS, and incubated for one extra hour at 37 °C in the presence of 1 mg/mL proteinase K. Subsequently, samples were extracted with 1 vol phenol-chloroform mixture and were loaded on a 1% agarose gel containing 0.1 μg/mL ethidium bromide. The gel was run for 16 h at 4 V/cm, and the images were acquired using a G-Box system (Syngene, United Kingdom).

### 2.6 Cell cycle analysis

One million promastigotes untreated or treated with 1 μM of different indenoisoquinolines were harvested by centrifugation after 0, 8, 24 and 48 h exposure. Promastigotes were washed twice with PBS and fixed by 1 h incubation with 70% methanol at 4 °C. After incubation, cells were centrifuged, washed again with PBS and resuspended in 0.5 mL of citrate buffer (45 mM MgCl_2_, 20 mM MOPS, 30 mM sodium citrate, 0.1% Triton X-100, pH = 7). Finally, samples with RNAase (50 μg/mL) and propidium iodide (5 mg/mL) were incubated at 37 °C during 30 min and analyzed at 589 nm by flow cytometry (Cyan ADP Dako).

### 2.7 Western blotting analyses

To detect H2A phosphorylation by *L. infantum* promastigotes, parasites in exponential growing phase (5–6 x 10^6^ cells/mL) were incubated at 26 °C with CPT derivatives and indenoisoquinolines at a final concentration in the supplemented 199 standard medium of 10 μM. Control cells were treated with the drug vehicle DMSO using concentrations below 0.1%. After 0, 15, 60 and 120-min incubations, promastigotes were harvested by centrifugation, washed twice in PBS and the pellet resuspended in lysis buffer containing 50 mM Tris (pH:7), 50 mM NaCl, 20 mM NaF, 0.1% Tween 20 and 2x protease inhibitors (PIERCE™ protease inhibitor mini tablets). Twenty micrograms of the different extracts were loaded onto 15% SDS-PAGE gels, electrophoresed and transferred to PVDF membranes (Immobilon-FL, Millipore) using a mini trans-blot electrophoretic transfer cell (Bio-Rad). Polyclonal γH2A antisera were used at a 1:2000 dilution and secondary goat anti-rabbit IgG IRDye680 (Li-Cor Biosciences) was used at a 1:15000 dilution. The peptide competition assay was done incubating the polyclonal γH2A antisera with 50 ng/mL of the appropriate peptide (with and without phosphorylation at Thr residue) in blocking buffer for 1 h at room temperature before blotting. Loading control was performed using a monoclonal α tubulin mouse antibody at 2 μg/mL (final concentration) and a goat anti-mouse IgG IRDye 800 (Li-Cor Biosciences) as secondary antibody. Bands were visualized in an Odissey® (Li-Cor Biosciences) infrared detection facility. The quantification of γH2A signal was done with Bio-Rad Quantity One Analysis Software.

### 2.8 Confocal microscopy and foci counting

γH2A detection and imaging were carried out as described previously. Control and treated promastigotes were collected and washed in PBS, seeded in μ-Slide 8 well (Ibidi GmbH, Germany) coated with poly-L-lysine and fixed for 30 min in 4% (v/v) paraformaldehyde on ice. Cells were washed 4x with PBS and then were incubated at −20 °C in 100% ethanol in order to permeabilize cells. Promastigotes were rehydrated 10 min in PBS. Blocking step was carried out in BSA 1% (w/v). Primary γH2A antibody and secondary fluorescein isothiocyanate (FITC) conjugated goat anti-rabbit (Pierce) were used at 1:500 and 1:100 dilution respectively. DNA was stained with 4,6-diamino-2-phenylindole (DAPI) prior to confocal microscopy. Observation and image acquisition of promastigotes were then taken at 488 nm using a Zeiss LSM800 confocal microscope. Foci counting was performed using Focinator (Oeck et al., 2015).

### 2.9 SDS/K-DNA precipitation

In order to measure the fragmentation of DNA induced by Top poisons, *L. infantum* promastigotes, previously labeled with 0.5 mCi/mL [2–^14^C] thymidine for 24 h, were exposed to different concentrations of indenoisoquinolines for 30 min, followed by precipitation with SDS/KCl, according to the method described previously (Balaña-Fouce et al., 2012). DNA fragmentation was determined as the percentage of total labeled DNA, and was calculated as follows: [(dpm in SDS/KCl drug - dpm in SDS/KCl solvent)/(dpm total incorporation)] x 100. Each experiment was run at least in triplicate.

## 3 Results

### 3.1 *In vitro* screening of indenoisoquinolines activity and TopIB inhibition

We have screened the antileishmanial effect of a small collection of 20 c indenoisoquinolines structure developed by Dr. Mark Cushman at Purdue University (USA), on the two biological forms of *L. infantum*. The antileishmanial activity of these compounds was assessed using the iRFP *L. infantum* strain, a genetically modified cell line able to constitutively produce the iRFP protein when viable. The advantage of this strain lies in the fact that the viability of both free-living and intra-macrophagic parasites exposed to different concentrations of a drug can be ascertained by measuring the infrared fluorescence emitted by the viable parasites, thus enabling the calculation of the EC_50_ values.

The compounds containing different substituents on the indenoisoquinoline scaffold were classified according to the functional moieties present at position N-6 of the indenoisoquinoline core. Table 1, Table 2, Table 3 include the leishmanicidal activity on the two forms of the parasite, the cytotoxicity in primary cultures of mouse splenocytes and the inhibition on recombinant hTopIB and LTopIB enzymes. Mouse splenocytes have been chosen because they naturally harbor the amastigote form of the parasite and, therefore, they are the cell line initially exposed to the potential toxicity of the drug. In addition, they are non-malignant cells and, in general, they are less susceptible to topoisomerase-targeting agents than cancer cells (Pantazis et al., 1993).

**Table 1:**
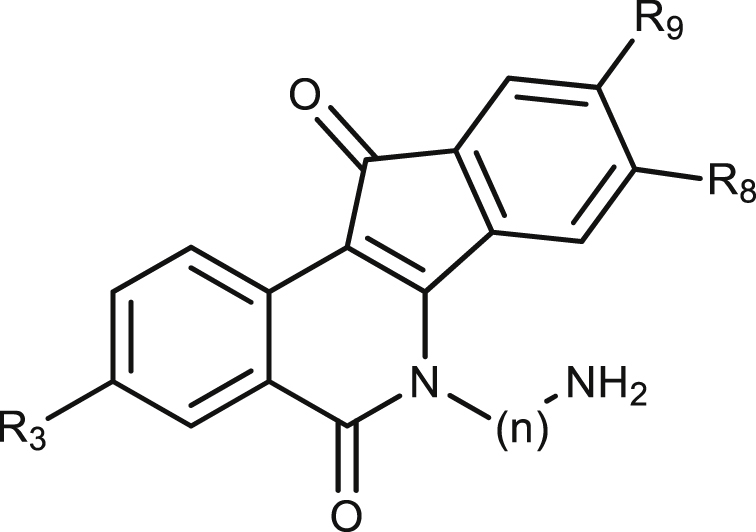
Bioactivity of *N*-6-aminoalkyl indenoisoquinolines on iRFP-*L. infantum* promastigotes and splenic-infecting amastigotes. The cytotoxic effect was assessed on mouse non-infected splenocytes using the Alamar Blue method. Each point represents the average of three different experiments by duplicate.

Cod. Purdue	Compound	n	R3	R8	R9	EC_50_ (μM)		CC_50_ (μM)	SI	LTopIB inhibition	hTopIB inhibition
						*L. infantum* promastigotes	*L. infantum* amastigotes	Murine Splenocytes			
**TN-1-61**	1	2	H	H	H	0.94 ± 0.03	0.04 ± 0.09	1.10 ± 0.06	24.4	+++	++
**TN-1-62**	2	3	H	H	H	0.34 ± 0.03	0.12 ± 0.05	0.59 ± 0.28	4.9	+++	++
**AM-14**–**58**	3	3	-NO_2_	H	-OCH_3_	0.20 ± 0.01	>10	0.41 ± 0.19	–	++	+++
**AM-14**–**67**	4	3	-NO_2_	H	-CO_2_CH_3_	1.10 ± 0.07	0.14 ± 0.08	0.59 ± 0.02	4.2	++	+++
**AM-14**–**32**	5	3	-NO_2_	H	-OCH_2_CH_3_	0.54 ± 0.05	0.08 ± 0.00	0.35 ± 0.03	4.4	++	+++
**AM-10**–**30**	6	3	-NO_2_	H	Br	0.52 ± 0.03	0.13 ± 0.03	0.81 ± 0.09	6.2	+++	+++
**AM-12**–**21**	7	3	-NO_2_	H	F	1.28 ± 0.08	0.04 ± 0.00	0.30 ± 0.07	8.4	++	+++
**AM-4-42**	8	3	-NO_2_	-OCH_2_O-	*	0.06 ± 0.01	0.02 ± 0.05	0.05 ± 0.01	2.8	+++	+++
**TN-1-65**	9	4	H	H	H	0.43 ± 0.08	0.73 ± 0.01	2.11 ± 0.22	2.4	+++	+++
**TN-1-75**	10	5	H	H	H	2.40 ± 1.03	2.29 ± 1.05	1.35 ± 0.19	–	+++	+++
**AMB**		Other				0.80 ± 0.10	0.30 ± 0.00	>20	>62	n/a	n/a

SI: Selectivity Index = CC_50_/EC_50_ (amastigotes).

*: –OCH_2_O- bridged positions R8 and R9.

+++: inhibition at 1 μM; ++: inhibition at 10 μM; +: inhibition at 100 μM; 0: no inhibition: n/a: no applicable.

**Table 2:**
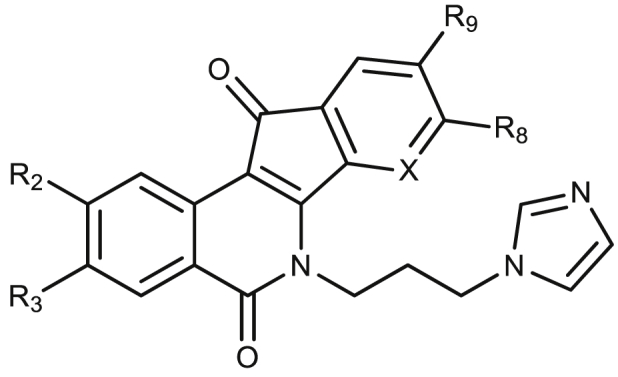
Bioactivity of *N*-6-imidazolylpropyl indenoisoquinolines on iRFP-*L. infantum* promastigotes and splenic-infecting amastigotes. The cytotoxic effect was assessed on mouse non-infected splenocytes using the Alamar Blue method. Each point represents the average of three different experiments by duplicate.

Cod. Purdue	Compound	R2	R3	R8	R9	X	EC_50_ (μM)		CC_50_ (μM)	SI	LTopIB inhibition	hTopIB inhibition
							*L. infantum* promastigotes	*L. infantum* amastigotes	Murine Splenocytes			
**AM-10**–**57**	11	H	-NO_2_	H	H	C	0.45 ± 0.50	0.06 ± 0.02	0.71 ± 0.01	11	+++	+++
**AM-14**–**19**	12	H	-NO_2_	H	-OCH_3_	C	>10	0.01 ± 0.00	0.33 ± 0.05	33	++	+++
**EK-4-41**	13	H	H	–	H	N	2.89 ± 0.14	0.59 ± 0.60	2.40 ± 0.35	4.1	+	++
**MNR-1-38**	14	-OCH_3_	-OCH_3_	-OCH_2_O-	*	C	1.85 ± 0.18	0.01 ± 0.00	0.03 ± 0.00	3.0	0	+++

SI: Selectivity Index = CC_50_/EC_50_ (amastigotes).

*: –OCH_2_O- bridged positions R8 and R9.

+++: inhibition at 1 μM; ++: inhibition at 10 μM; +: inhibition at 100 μM; 0: no inhibition.

**Table 3:**
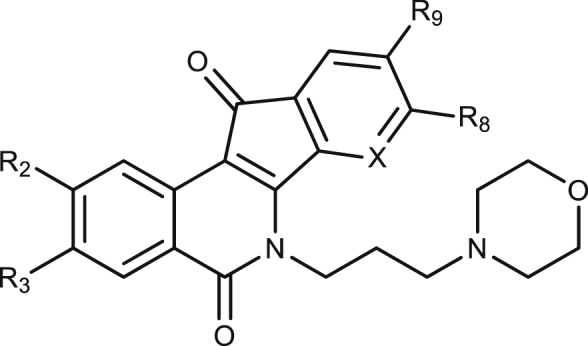
Bioactivity of *N*-6-morpholinopropyl indenoisoquinolines on iRFP-*L. infantum* promastigotes and splenic-infecting amastigotes. The cytotoxic effect was assessed on mouse non-infected splenocytes using the Alamar Blue method. Each point represents the average of three different experiments by duplicate.

Cod. Purdue	Compound	R2	R3	R8	R9	X	EC_50_ (μM)		CC_50_ (μM)	SI	LTopIB inhibition	hTopIB inhibition
							*L. infantum* promastigotes	*L. infantum* amastigotes	Murine Splenocytes			
**AM-10**–**63**	15	H	-NO_2_	H	H	C	>10	0.60 ± 0.42	17.99 ± 5.30	30	0	+++
**AM-14**–**16**	16	H	-NO_2_	H	-OCH_3_	C	>10	1.86 ± 0.29	0.59 ± 0.08	–	0	+++
**EK-5-9**	17	-OCH_3_	H	–	-OCH_3_	N	2.92 ± 0.44	0.27 ± 0.08	7.84 ± 0.77	29.0	+++	+++
**EK-5-71**	18	-OCH_3_	-OCH_3_	–	-OCH_3_	N	0.53 ± 0.07	0.13 ± 0.01	3.81 ± 0.51	29.3	++	++

SI: Selectivity Index = CC_50_/EC_50_ (amastigotes).

+++: inhibition at 1 μM; ++: inhibition at 10 μM; +: inhibition at 100 μM; 0: no inhibition.

The results in Table 1 show the bioactivity of *N*-6 aminoalkyl indenoisoquinolines, where the chain-length effects of the substituents at position *N*-6 (n = 2 to n = 5), along with the presence of other groups at positions C-3, C-8 and C-9, were assessed. The antileishmanial effect of most of these compounds was within the micromolar or submicromolar range. Such potency is similar to that of CPT (EC_50_ = 0.03 μM in amastigotes) (Prada et al., 2013) and to the antileishmanial drug AMB (EC_50_ = 0.3 μM in amatigotes) in clinical use (Escudero-Martínez et al., 2017). Furthermore, all the compounds of this series were able to inhibit the relaxation activity of both recombinant hTopIB and LTopIB at 10 μM or less. When the killing effects of compounds **1**, **2**, **9** and **10**, which have similar substituents at positions C3, C8 and C9 but a variable chain length (n) at *N*-6 (2–5, respectively), were compared, it was found that the shorter the length of the chain, the stronger the antileishmanial effect, especially against the intramacrophagic amastigotes (the most relevant clinical form). In this case, compound **1** was the most effective with an EC_50_ = 0.04 μM. On the other hand, when we compared the cytotoxic effects of the compounds in non-infected splenocytes exposed to this series of indenoisoquinolines, compound **2** exhibited the maximum cytotoxic effect on the mammalian cell culture, unlike compound **1** which showed an interesting SI = 24.4.

The antileishmanial potencies of two series of *N*-6-imidazolylpropyl (Table 2) and *N*-6-morpholinopropyl (Table 3) indenoisoquinolines were also assessed. The replacement of the NH_2_-terminal of the aminoalkyl moiety at position N-6 by imidazole or morpholino rings reduced the potency in killing promastigotes (compare compounds **3**, **12** and **16**), but not amastigotes or non-infected splenocytes. Consequently, the SI of compound **12** improved to an interesting value of 33. On the other hand, compounds **17** and **18** were among those with the best selectivity indexes (SI = 29 and SI = 29.3, respectively) in this study.

### 3.2 Cell cycle analysis

**Fig. 1:**
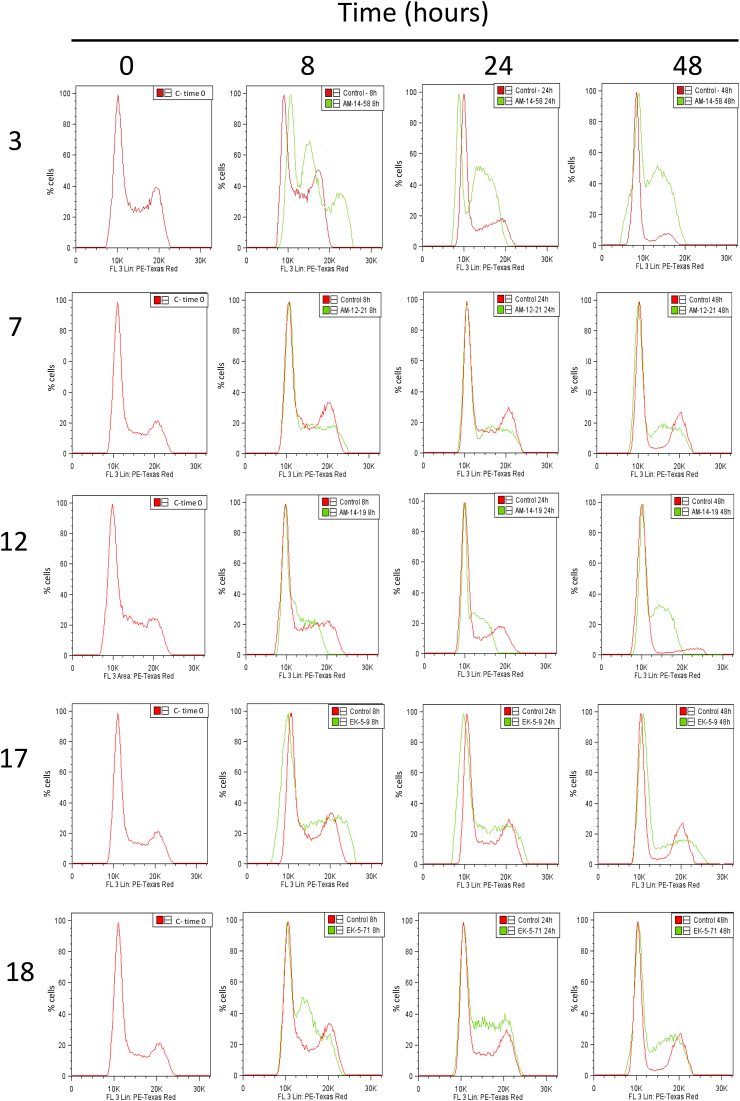
**Cell cycle proliferation of free-living *L. infantum* promastigotes.** Cells treated with a single dose of indenoisoquinoline (green line) or without treatment (red line) were fixed and stained with propidium iodide and analyzed at the indicated times by flow cytometry. (For interpretation of the references to colour in this figure legend, the reader is referred to the Web version of this article.)

We studied the effects of these indenoisoquinolines on the leishmanial cell cycle by adding a single high dose of each compound to free-living promastigotes. Fig. 1 shows the effects on the *L. infantum* cell cycle of a group of compounds selected from Table 1, Table 2, Table 3 that strongly inhibited LTopIB. This was analyzed by flow-cytometry at 0 h, 8 h, 24 h and 48 h post-treatment. Compounds **3** and **7**, which belong to the N-6-aminoalkyl indenoisoquinolines group, arrested the cell cycle of promastigotes in S phase. This arrest was maintained until the end of the time evaluated. Similar results were obtained with *N*-6-imidazolylpropyl indenoisoquinoline **12** and *N-*6-morpholinopropyl indenoisoquinolines **17** and **18**.

### 3.3 H2A phosphorylation in *Leishmania*

The stabilization of transient DNA-TopIB cleavage complexes by Top poisons - such as CPT and indenoisoquinolines - may lead to their collision with DNA replication forks, causing DSBs (Staker et al., 2005). Post-translational modifications of histones facilitate the access of DNA-repair enzymes to nucleosomes. The phosphorylation of Ser^139^ in H2AX histones (γH2AX) is one of the earliest responses to DSB generation, and has been used as damage biomarker to monitor the activity of Top poisons in human cells (Rothkamm and Löbrich, 2003; Huang et al., 2003).

**Fig. 2:**
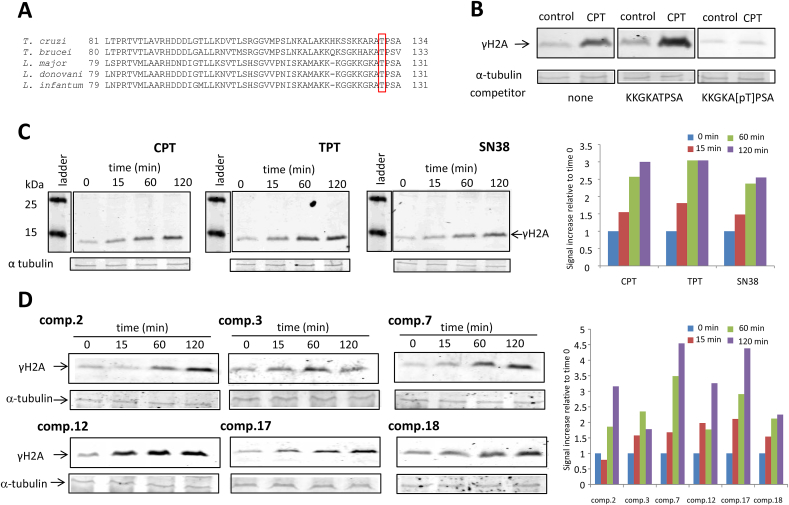
**Histone H2A is phosphorylated in *L. infantum* promastigotes as response of LTopIB poisons. A)** Partial alignment of the C-terminal end of trypanosomatid's histone H2A, showing the Thr residue suitable for phosphorylation as response of DNA damage. **B)** Peptide competition assay performed to demonstrate the specificity of the antibody prepared against KKGKA [pT]PSA. γH2A signal induced in cells treated with CPT (10 μM) decreased when the antibody competed with the phosphorylated peptide. **C)** Time-course phosphorylation of histone H2A analyzed by Western-blot hybridization with the polyclonal antibody, of promastigotes exposed to 10 μM CPT (left), TPT (middle) and SN38 (right). The histogram shows the signal increase of γH2A relative to time 0. **D)** Time-course phosphorylation of histone H2A in promastigotes exposed – from left to right – to compounds **2**, **3**, **7**, **12**, **17** and **18**. *L. infantum* promastigotes were incubated with the reagents for 15, 60 and 120 min. Alfa-tubulin band is used as housekeeping control. The histogram shows the signal increase of γH2A relative to time 0.

Trypanosomatids have a histone H2A that lacks the characteristic SQ-motif involved in the phosphorylation site of eukaryotic H2AX histones. Glover and Horn (2012) described a Thr residue in this position in the unfolded tail of *T. brucei* H2A (Thr-130) suitable for phosphorylation in response to DNA damage. In *Leishmania*, the Thr-128 residue placed at the C-terminal end of the H2A histone could play the role of an unusual phosphorylation motif (Fig. 2A) and has previously been used as a marker of DNA damage in *L. major* (Damasceno et al., 2016, 2018). To address the possibility of H2A phosphorylation in response to LTopIB poisons in *Leishmania* promastigotes, a polyclonal γH2A antiserum was prepared using the phosphorylated KKGKA [pT]PSA peptide as antigen. Thereby, the ability of *L. infantum* promastigotes to phosphorylate histone H2A at this amino acid in response to CPT, SN38 and TPT (known TopIB poisons inducers of γH2AX signaling in human cells (Huang et al., 2003), was assessed by western-blot. Promastigotes in exponentially growing phase were incubated with the selected compounds at a fixed concentration of 10 μM in the standard culture medium, and aliquots were harvested after 0, 15, 60 and 120 min. Phosphorylation of H2A was addressed by Western blot analysis using the polyclonal antibody described and a secondary antirabbit IRDye 680 antibody. Fig. 2C is a representative time-course experiment that shows a clear increase in the intensity of the 14-kDa band, which corresponds to γH2A protein, in response to the added compounds.

According to these results, phosphorylation of H2A histone on Thr^128^ was used as a DSB biomarker in response to the selected indenoisoquinolines with stronger LTopIB inhibition. Fig. 2D shows the compounds that induced a substantial increase in γH2A levels.

### 3.4 γH2A foci generation in *Leishmania* promastigotes

**Fig. 3:**
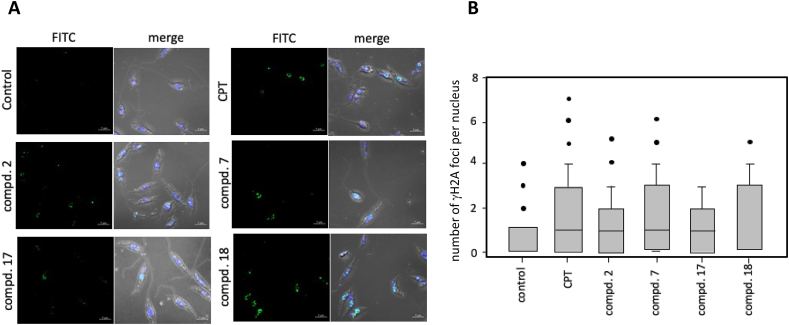
**γH2A foci formation as response to CPT and indenoisoquinolines. A)** Confocal microscopy images showing histone γH2A phosphorylation as response to CPT and **2**, **7**, **17** and **18** indenoisoquinolines for a period of 30 min. Images were acquired in a Zeiss LS800 confocal microscope using 100× magnification. DNA was stained with DAPI and γH2A with specific primary and secondary (FITC labeled) antibodies. Scale bar = 5 μm. **B)** Box-plot distribution values representing the number of foci per nuclei obtained for each drug treatment. The foci number was obtained using the macro “Focinator” in the open-source program ImageJ. Two hundred cells were analyzed in two independent experiments for each treatment.

To corroborate H2A phosphorylation in response to TopIB inhibitors, we proceeded to visualize the generation of γH2A foci around DSBs by immunofluorescence microscopy (Furuta et al., 2003). To this end, *L. infantum* promastigotes were exposed to a single dose of CPT and the indenoisoquinolines **2**, **7**, **17** and **18**, to evaluate the generation of γH2A foci for a period of 2 h (Fig. 3A). Fig. 3B shows the results obtained by scoring those cells that had at least 2 foci inside the nucleus positive for γH2A. Both CPT and indenoisoquinolines showed statistically significant differences (p < 0.05) in a *t*-Test analysis with respect to untreated cells.

### 3.5 5*. In situ* cleavage complex formation

**Fig. 4:**
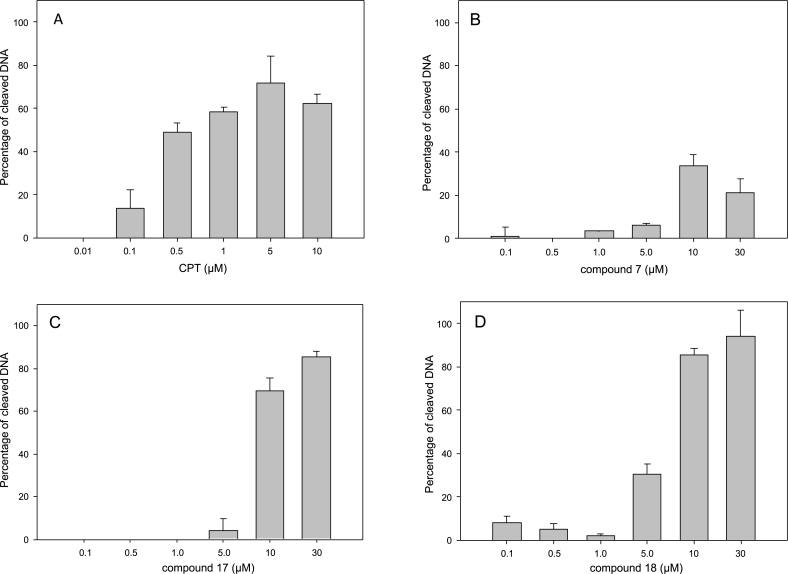
**Induction of SDS/K-DNA precipitable complexes by indenoisoquinolines in *L. infantum* promastigotes.** CPT (A) – as positive control – and the indenoisoquinolines **7** (B), **17** (C) and **18** (D) were added to cultures of *L. infantum* promastigotes, previously labeled with 0.5 mCi/mL [2–^14^C] thymidine for 24 h, for 30 min at the concentrations indicated in the bars. After this time, the percentage of SDS/K-DNA precipitable complexes was determined. Results are expressed as means ± SE for at least three different experiments carried out in duplicate.

Bakshi and coworkers indicated that the stabilization of TopIB-DNA complexes by a battery of indenoisoquinolines was not the only origin of the killing mechanism of these compounds in African trypanosomes, which did not correlate with their ability to trap cleavage complexes (Bakshi et al., 2009). Thus, we studied the stabilization of cleavage complex *in situ* produced by the indenoisoquinolines previously chosen (due to their ability to inhibit LTopIB and to induce H2A phosphorylation). This was tested by the formation of SDS-precipitable enzyme–DNA adducts. With this purpose, *L. infantum* promastigotes were grown in the presence of [2–^14^C]-thymidine and exposed to increasing concentrations of each selected compound over a period of 30 min. CPT was used as reference drug due to its ability to generate TopIB-DNA complexes in *Leishmania* parasites as previously reported (Prada et al., 2013) (Fig. 4).

The 7-azaindenoisoquinolines **17** and **18** showed the maximum DNA-cleaving potency, and reached similar levels to that found for CPT at the highest final concentration of 10 μM. However, compound **2**, which was one of the strongest compounds in preventing supercoiled DNA relaxation by LTopIB, hardly produced perceptible protein-DNA complexes (10%) at the highest concentration (30 μM) (data not shown). The maximum percentage obtained with indenoisoquinoline **7** was 33%, a value that was far from the percentages obtained with the reference drug.

## 4 Discussion

Indenoisoquinolines belong to a family of non-CPT compounds that were initially synthesized as anticancer drugs, and whose mechanism of action is based on TopIB poisoning. Previous drug-repurposing studies showed that some of these compounds have a potent trypanocidal effect *in vitro* and *in vivo* against both *T. brucei* (Bakshi et al., 2009) and *L. infantum* (Balaña-Fouce et al., 2012; Prada et al., 2013), thus promoting their potential development as antiparasitic drugs.

The battery of indenoisoquinolines studied in this work emphasizes mainly the modifications in the *N*-6 position of the heterocyclic system, which has been shown to generate better inhibitors of TopIB with antitumor effects (Morrell et al., 2007) and improved trypanocide activity (Bakshi et al., 2009). The results obtained in this study indicate that the LTopIB inhibition profile of most compounds hardly correlates with the SI values. The best SI values were observed with *N*-6-imidazolylpropyl and *N*-6-morpholinopropyl derivatives, compounds **12**, **15**, **17**, and **18**. These results agree well with those obtained previously with indotecan and AM13-55, two *N*-6-morpholinopropyl indenoisoquinolines that showed interesting therapeutic potential against *L. infantum* VL *in vivo* (Balaña-Fouce et al., 2012). It is remarkable that among N-6-morpholinopropyl derivatives, those including an N atom in the indenoisoquinoline ring (7-azaindenoisoquinolines, compounds **17** and **18**) had the highest LTopIB inhibitory activity. It has been described that the incorporation of an N atom in the heterocyclic system of these molecules increases their ability to stabilize the ternary cleavage complex by interacting with neighboring DNA bases (Kiselev et al., 2011).

Similar to other TopIB poisons, indenoisoquinolines arrest cell cycle progression in both S and G2-M phases in human cancer cells, with the phosphorylation of H2AX taking place mainly in S-phase (Antony et al., 2007). The cell cycle analysis of *L. infantum* promastigotes exposed to indenoisoquinolines with LTopIB inhibitory activity showed that not all of these compounds were able to arrest the cell cycle. However, the compounds that produced S-phase arrest also showed an increase in γH2A, excluding the indenoisoquinoline **2**, which induced strong γH2A signaling and did not arrest the cell cycle.

The ability of these compounds to trap adducts did not correlate with their ability to inhibit LTopIB *in vitro* or to phosphorylate H2A. This is similar to what it occurs with the correlation of cleavage-complex formation and the killing ability of indenoisoquinolines in *T. brucei* (Bakshi et al., 2009). The higher capacity of 7-azaindenoisoquinolines to trap this complex (compounds **17** and **18**) compared with compounds **2** and **7**, which inhibit LTopIB and produce H2A phosphorylation, could be explained by the inhibitory effect over tyrosyl DNA-phosphodiesterase (Tdp1) of 7-azaindenoisoquinolines, a key DNA-repair enzyme. This enzyme is involved in removing a variety of adducts bound to 3′-DNA, including TopIB-DNA complex (Dexheimer et al., 2008; Huang et al., 2011), and its presence has been described in *L. donovani* (Banerjee et al., 2010). Initially, 7-azaindenoisoquinolines were synthesized as dual TopIB/Tdp1 inhibitors (Wang et al., 2017), and the high rates of DNA fragmentation provided by these compounds and associated with high histone H2A phosphorylation and cell cycle arrest, may be attributed to a poor repair capacity after DNA injury.

In conclusion, the antileishmanial effect of indenoisoquinolines involves several mechanisms different from TopIB inhibition. Since stabilization of the cleavage complex does not correlate with DSB generation, other processes that are able to induce or repair this process are likely to be involved.

## Author contributions

YPP, RBF and RRT conceived and designed the study. YPP and CGE drafted the manuscript and YPP, CGC and RAV performed the experiments and analyzed the data. MC provided indenoisoquinolines from his collection and edited the manuscript. All authors read and approved the final draft.
